# Exploring the Mechanisms Underlying the Phenomenon of Inner Speaking-Induced Suppression

**DOI:** 10.1523/ENEURO.0047-26.2026

**Published:** 2026-07-24

**Authors:** Thomas J. Whitford, Marianthe Godwin, Damien J. Mannion, Lawrence Kin-hei Chung, Kevin M. Spencer, Yoji Hirano, Oren Griffiths, Wadim Vodovozov, Anthony W. F. Harris, Mike E. Le Pelley, Bradley N. Jack

**Affiliations:** ^1^School of Psychology, University of New South Wales (UNSW Sydney), Sydney, New South Wales 2052, Australia; ^2^Brain Dynamics Centre, Westmead Institute for Medical Research, Sydney, New South Wales 2145, Australia; ^3^Melbourne Data Analytics Platform (MDAP), University of Melbourne, Melbourne, Victoria 3010, Australia; ^4^Department of Psychology, The Chinese University of Hong Kong, Sha Tin, New Territories, Hong Kong SAR 999077, China; ^5^Veterans Affairs Boston Healthcare System, Boston, Massachusetts 02130; ^6^Department of Psychiatry, Harvard Medical School, Boston, Massachusetts 02138; ^7^Department of Psychiatry, Division of Clinical Neuroscience, Faculty of Medicine, University of Miyazaki, Miyazaki 889-1692, Miyazaki Prefecture, Japan; ^8^School of Psychology, University of Newcastle, Newcastle, New South Wales 2308, Australia; ^9^Department of Psychiatry, Zucker Hillside Hospital, Glen Oaks, New York, New York 11004; ^10^Research School of Psychology, Australian National University, Canberra, ACT 2600, Australia

**Keywords:** auditory-evoked potential, corollary discharge, EEG, efference copy, inner speech

## Abstract

Speaking-induced suppression (SIS) refers to the phenomenon that the sounds produced through self-generated speech elicit less EEG activity in the auditory cortex than externally produced sounds. An analogous phenomenon demonstrates that the activity elicited by an audible syllable is suppressed when it co-occurs with a content-matching syllable produced in inner speech. We have argued that this “inner SIS” effect is likely underpinned by corollary discharges, similar to those assumed to underlie SIS. However, an alternative possibility is that “inner SIS” reflects a priming effect, wherein the increased subjective expectation of hearing a certain sound causes a reduction in evoked activity. The current study tested this alternative hypothesis using an expectancy-only version of the task in which participants (*N* = 70, 45 female and 25 male) did not produce inner speech. On each trial, a syllable appeared on-screen (/ba/, /bi/, or /fo/) and was followed by an audible syllable (/ba/ or /bi/). The on-screen syllable either matched (Primed condition) or mismatched (Misprimed condition) the content of the audible syllable. In the Control condition, the on-screen syllable never occurred as the audible syllable. Contrary to previous studies in which participants produced inner speech, there were no significant between-condition differences in N1 amplitude, P2 amplitude, prestimulus activity, or gamma-band power elicited by the audible syllable. Bayesian analysis indicated a meaningful difference between these findings and prior results from the inner-speech task. The present data indicate that a simple cue-based priming manipulation, in the absence of inner speech, is insufficient to reproduce previously reported inner SIS effects.

## Significance Statement

Inner speaking-induced suppression (“inner SIS”) refers to the phenomenon that the electrophysiological activity elicited by an external vocalization is reduced when participants concurrently produce the same vocalization in their inner speech. We, and others, have argued that inner SIS is likely caused by corollary-discharge–related mechanisms. However, an alternative possibility is that inner SIS reflects a priming effect, wherein the increased subjective expectation of hearing a certain vocalization causes a reduction in evoked activity. Using a modified version of our standard experimental protocol, we found no evidence for this alternative hypothesis. This result suggests that a priming manipulation, in the absence of inner speech, is insufficient to reproduce previously reported inner SIS effects.

## Introduction

Speaking-induced suppression (SIS) refers to the phenomenon that the sounds produced through self-generated speech elicit less activity in the auditory cortex than externally produced sounds, even when then the sounds themselves are physically identical (Houde et al., 2002; Heinks-Maldonado et al., 2005; Ventura et al., 2009; Niziolek et al., 2013; Whitford, 2019). SIS is often operationalized as a reduction in the amplitude of the N1/M100 component of the auditory-evoked potential to self-generated versus externally generated speech (Curio et al., 2000; Sitek et al., 2013). SIS is believed to be caused by an efference copy of speaking-related motor commands, which is used to make a neural prediction (corollary discharge) of the expected sensory consequence of the speech sounds (Niziolek et al., 2013; Eliades and Wang, 2019; Whitford, 2019). The corollary discharge is thought to predict and suppresses the neurophysiological activity elicited by the speech sound to the extent that the predicted sensory feedback matches the actual sensory feedback (Behroozmand et al., 2009; Behroozmand and Larson, 2011).

While the phenomenon of SIS refers to the production of overt speech, an analogous phenomenon has been identified in the context of inner speech (Tian and Poeppel, 2010, 2013, 2015; Whitford et al., 2017, 2025a,b; Jack et al., 2019; Li et al., 2020; Berryman et al., 2025), which refers to the silent production of words in one's mind (Perrone-Bertolotti et al., 2014; Alderson-Day and Fernyhough, 2015). The experimental protocol we have developed to assess SIS to inner speech can be summarized as follows: the participant is asked to produce a designated inner syllable (either /ba/ or /bi/) at a precisely defined time indicated by means of visual cue. At the exact same time an audible syllable is delivered (via headphones) of a speaker producing either the syllable /ba/ or the syllable /bi/. There are three conditions: trials in which the inner and audible syllables match on content (Match condition), trials in which they mismatch on content (Mismatch condition), and trials in which the participant does not produce an inner syllable but still hears the audible syllable (Passive condition). Using this task, we have previously observed a reduction in the amplitude of the N1 amplitude in the Match condition, relative to both the Passive and Mismatch conditions. We have dubbed this finding “inner speaking-induced suppression” or “inner SIS” (Whitford et al., 2017, 2025b; Jack et al., 2019).

We have suggested that the phenomenon of “inner SIS” is likely to be caused by a corollary discharge or related mechanism, in much the same way that corollary discharges are believed to underlie the phenomenon of SIS in the context of overt speech (Tian and Poeppel, 2013; Whitford et al., 2017; Jack et al., 2019; Whitford, 2019). This suggestion is consistent with the theory that inner speech is ultimately a “special type” of overt speech in which the articulator organs do not move (Feinberg, 1978; Jones and Fernyhough, 2007). However, an alternative possibility raised by Patel (Patel, 2022) argues that the “inner SIS” effect may not be caused by a corollary discharge and indeed not caused by inner-speech production at all but may rather reflect a more general expectancy effect. Specifically, Patel proposed that when the participant is preparing to produce an inner syllable (e.g., /ba/), this “primes” the sound in their mind such that when the sound /ba/ is actually heard, the N1 amplitude elicited by the sound is reduced due to the fact that it was subjectively expected*.* While it is important to note that the content of the inner syllable is never objectively predictive of the audible syllable (as the audible syllable is equally likely to be /ba/ or /bi/ on any given trial), Patel argues that it could nonetheless be subjectively predictive: “Subjects were cued to produce either /ba/ or /bi/ at the outset of each run and the objective probability that the cued speech sound would be presented was .5. Within this unpredictable environment, I suggest that cueing subjects to produce a speech sound primes them to expect that speech sound to occur, resulting in the attenuation of the speech sound that matches the cue-based expectation” (Patel, 2022, p. 11).

This explanation, if correct, would substantially alter our interpretation of the inner SIS effect; indeed, the very term “inner SIS” would be a misnomer, as it would imply the effect was not caused by inner-speech production at all. The aim of the current study was thus to investigate whether the phenomenon of “inner SIS” could be elicited by a task in which participants were “primed” to hear certain audible syllables but, crucially, were not instructed to produce inner speech. We also investigated whether the various other ERP and oscillatory changes that we have previously observed in our inner-speech task—namely, changes in the P2 component (Whitford et al., 2017; 2025b), prestimulus activity (Chung et al., 2023), and spectral power in the gamma (30–100 Hz) and sub-gamma frequency bands (Whitford, et al., 2025b)—were also apparent in this modified design of the experimental task.

## Materials and Methods

### Participants

Seventy-three undergraduate students participated in exchange for course credit. Three participants were excluded for either failing to complete the task (one participant) or for having excessive artifact in their electroencephalographic recording (two participants)—see below, EEG processing and analysis, for more details. This left a final sample of 70 participants; mean age was 20 years (SD = 3.3), 45 were female (64%), and 65 were right-handed (93%). The study was approved by the Human Research Ethics Advisory Panel (Psychology).

### Apparatus, stimuli, and procedure

Participants were seated in a quiet, dimly lit room, ∼60 cm from a computer monitor (BenQ XL2420T, 1,990 × 1,080 pixels, 64 cm diagonal) and were fitted with over-ear headphones (AKG K77 Perception). Stimulus presentation was controlled via MATLAB using the Psychophysics Toolbox (Brainard, 1997; Kleiner et al., 2007).

The experimental protocol was a modified version of the task which we have previously used to investigate inner speech (Whitford et al., 2017; 2025b; Chung et al., 2023). There were three separate block types (“Prime /ba/,” “Prime /bi/,” and “Prime /fo/”), and each block was preceded by instructions provided on the screen. In “Prime /ba/” blocks, participants were given the following instructions at the start of the block: “In this block, we want you to simply sit back, relax, and listen to the sounds while keeping your eyes fixated on the stationary vertical red line. At the precise moment the moving vertical green line overlaps with the stationary red line, a sound will be played through your headphones. The sound will be of a male voice either saying the word BA, or saying the word BI. After every trial you will be asked to whether you followed instructions and paid attention on the trial. If you didn't, that's OK, just let us know and we will discard the trial. At the beginning of each trial, the word [XX] will be written on the screen. Please pay attention to what this word is. You will periodically be asked to remember what word was presented on the screen at the start of the trial” (Note that [XX] was replaced by BA in “Prime /ba/” blocks, BI in “Prime /bi/” blocks, and FO in “Prime /fo/” blocks).

**Figure 1.**
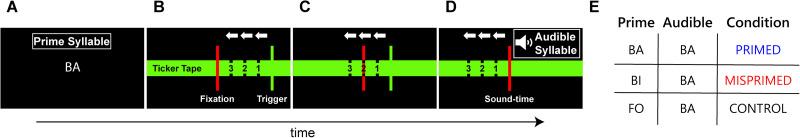
A schematic of the experimental protocol. Participants were first shown the prime syllable (“BA,” “BI,” or “FO”) and instructed to attend to and remember the syllable on the subsequent trial (Panel ***A***). After a delay of 2 s, the animation was presented on the screen (Panel ***B***). Participants were instructed to fixate their eyes on the central red fixation line, which was embedded in the thick green horizontal bar (the “tickertape”; see Panel ***A***). After a 1–2 s delay, the green trigger line, which was initially presented near the right-hand side of the screen, and visible in participants' peripheral vision, began to move slowly across the screen in a leftward direction at a speed of 6.5° of visual angle per second (see Panel ***C***), such that after ∼3.75 s, the green trigger line overlapped with the red fixation line. At this exact time, dubbed the “sound time” (Panel ***D***) an audible syllable was delivered through participants headphones. The audible syllable was of a male speaker producing the syllable BA or BI. In Primed trials (Panel ***E***, top), the primed syllable was the same as the audible syllable (e.g., prime /ba/, sound /ba/). In Misprimed trials (Panel ***E***, middle), the primed syllable was the same as the audible syllable that was not presented (e.g., prime /bi/, sound /ba/). In the Control condition, the primed syllable did not match either of the two possible audible syllables (e.g., prime /fo/, sound /ba/). Following the “sound time,” the trigger line continued to move leftward past the fixation line for an additional 1 s. The participant was then asked to rate how successfully they were able to follow the instructions on the trial by pressing “1” if they “successfully followed instructions and paid attention” and “9” if they “did not follow instructions or did not pay attention.”

Following the instructions, the experimental trials began. Every trial began with the primed syllable (i.e., /ba/, /bi/, or /fo/) being presented on the screen for 2 s (Fig. 1*A*). The animation display was then presented on the screen (*B*), consisting of a thick horizontal green line in the center of the screen (the “tickertape”), a thin vertical red line in the center of the screen (the “fixation line”), and a thin vertical green line which started near the right side of the screen (the “trigger line”). Participants were instructed to keep their gaze fixated on the fixation line (which remained stationary) for the duration of the trial. After a 1 s delay, the tickertape and its embedded trigger line began to move slowly leftward across the screen (C), at a speed of 6.5° visual angle/s, so that after 3.75 s the green trigger line intersected with the red fixation line (D). At the time of intersection, an audible syllable was played over the participant's headphones; this was a male speaker producing either the syllable BA or the syllable BI (∼70 dB SPL intensity and 200 ms duration); note that FO was never presented as an audible syllable.

After each trial, participants were asked to rate whether they had been able to follow the instructions on this trial, by pressing either “1” (“I successfully followed instructions and paid attention”) or “9” (“I did not follow instructions or did not pay attention”). Trials that were rated as “9” were excluded from the analysis.

On four randomly selected trials per block, participants were asked to report the syllable they were instructed to remember for that particular trial (which didn't change over the course of the entire block) and respond via the keyboard. This was to ensure that participants were attending to the task instructions. Each block consisted of 24 trials, and each of the three block types was presented a total of four times over the course of the experiment. The order of the blocks was randomized. Within each block, the audible syllable BA was presented on 12 trials and the audible syllable BI was presented on 12 trials, in a random order.

The data were parsed into three discrete trial types, which were analyzed as separate conditions (Fig. 1*E*): (1) **Primed** trials, in which the syllable the participant was asked to pay attention to and remember was the same as the one they heard (i.e., prime /ba/, hear /ba/ or prime /bi/, hear /bi/); (2) **Misprimed** trials, in which the syllable the participant was asked to pay attention to and remember was the other of the two possible audible syllables (i.e., prime /ba/, hear /bi/; or prime /bi/, hear /ba/); (3) **Control** trials, in which the participant was instructed to pay attention and remember a syllable (/fo/) that they never heard (i.e., prime /fo/, hear /ba/; or prime /fo/, hear /bi/). We chose the syllable /fo/ for the Control condition simply because it had similar properties to the two other syllables used in the task (/ba/ and /bi/); that is, it is a one-syllable, two-letter sound which is straightforward to pronounce and commonly used in English.

### EEG acquisition

EEG data were recorded with a BioSemi ActiveTwo system using 64 Ag/AgCl active electrodes placed according to the extended 10–20 system (FP1, FPz, FP2, AF7, AF3, AFz, AF4, AF8, F7, F5, F3, F1, Fz, F2, F4, F6, F8, FT7, FC5, FC3, FC1, FCz, FC2, FC4, FC6, FT8, T7, C5, C3, C1, Cz, C2, C4, C6, T8, TP7, CP5, CP3, CP1, CPz, CP2, CP4, CP6, TP8, P9, P7, P5, P3, P1, Pz, P2, P4, P6, P8, P10, PO7, PO3, POz, PO4, PO8, O1, Oz, O2, Iz). External electrodes were also placed on the left and right mastoid bones (the average of which were used for offline re-referencing), the tip of the nose, the outer canthus of the left and right eye (for the horizontal electrooculogram), and below the left eye (for the vertical electrooculogram, referenced to electrode Fp1). The sampling rate of the EEG was 2,048 Hz.

### EEG processing and analysis

The procedure for EEG preprocessing and analysis was based on the protocol described in Whitford et al. (2025b), and performed in BrainVision Analyzer (version 2.2, Brain Products). The data were bandpass filtered from 0.1 to 100 Hz using a phase-shift free Butterworth filter (Order 2), with a 50 Hz notch filter to minimize mains artifact. For the N1 and P2 analyses, the filtered data were separated into 600 ms epochs (200 ms prior to sound onset, 400 ms postonset), corrected for eye movements using the technique described in Gratton et al. (1983). We also used independent components analysis to identify and remove any headphone-related muscle artifact that occurred in the first 30 ms following on the onset of the audible syllable (Makeig et al., 1995). Any epoch with a signal exceeding a peak-to-peak amplitude of 200 µV was defined as unusable and excluded. As noted earlier, epochs were also classified as unusable if the participant reported that they “did not follow instructions or did not pay attention” on the trial. Two participants were removed for generating ≤25% of usable epochs in one or more conditions. For the remaining participants, there was an average of 89.09 (SD = 9.27) usable trials in the Primed condition, 88.89 (SD = 10.17) usable trials in the Misprimed condition, and 90.73 (SD = 9.20) usable trials in the Control condition. The number of usable epochs did not differ significantly between conditions (*F*_(2,138)_ = 3.464; *p* = 0.056). These usable epochs were baseline corrected to the mean voltage of the interval from −200 to 0 ms and were used to generate each participant's average ERP for the three conditions. Preprocessing for the analysis of the prestimulus activity was identical, except that the filtered data were separated into 2,600 ms epochs (2,500 ms prior to sound onset to 100 ms postonset) and baseline corrected to the average voltage in the window −2,500 to −2,000 ms (Chung et al., 2023).

For the time–frequency analysis, the analysis pipeline was based on the protocol described in Whitford et al. (2025b). In brief, a 1,050 ms epoch was extracted from the filtered data (−450 to 600 ms) and ocular correction, and artifact rejection was completed based on the procedure described above. A Morlet complex wavelet transformation was applied to each participant's average waveform from the Primed, Misprimed, and Control conditions. There were 40 logarithmically spaced frequency steps from 5 to 100 Hz, with wavelet center frequencies at 5, 5.399, 5.830, 6.296, 6.978, 7.341, 7.927, 8.560, 9.244, 9.982, 10.779, 11.639, 12.569, 13.572, 14.656, 15.826, 17.089, 18.454, 19.927, 21.518, 23.236, 25.091, 27.095, 29.258, 31.594, 34.116, 36.840, 39.782, 42.958, 46.388, 50.091, 54.091, 58.409, 63.073, 68.108, 73.546, 79.418, 85.759, 92.606, and 100 Hz. The Morlet parameter was set to 5. The output value was spectral power, in units of µV^2^. A decibel (dB) transform was applied to normalize power [10 × log 10 (power/baseline)]. The decibel transform removes scale differences between individuals, time points, and frequencies, rendering them more statistically comparable (Roach and Mathalon, 2008; Munneke et al., 2015). Baseline correction was applied per frequency band in the time window from −250 to −50 ms relative to the onset of the audible syllable. The epochs were clipped from −250 to 400 ms in order to remove edge artifacts.

### Statistical analysis

The ERP data were analyzed with a repeated-measures ANOVA, using SPSS Statistics 29 (IBM). There were two factors: Condition (three levels: Primed, Misprimed, and Control) and Electrode (nine levels for the analysis of the N1 and the P2; three levels for the analysis of the prestimulus activity). The electrodes-of-interest were centered on the electrode for which the relevant component was maximal in the collapsed localizer waveform (Luck and Gaspelin, 2017); the maximal electrode was FCz for the N1 component (thus the electrodes-of-interest for the N1 analyses were Fz, FCz, Cz, F1, F2, FC1, FC2, C1, C2) and Cz for the P2 component (thus the electrodes-of-interest for the P2 analysis were FCz, Cz, CPz, FC1, FC2, C1, C2, CP1, CP2), as per the procedure we used in our previously published protocol (Whitford et al., 2025b). For the analysis of the prestimulus activity, the maximal electrode was Cz, and the electrodes-of-interest were Cz, FCz, and CPz, as per the procedure used in our previous protocol (Chung et al., 2023).

There were three dependent variables in the ERP analysis, namely, the amplitude of the N1 component, P2 component, and prestimulus activity of the auditory-evoked potential. The amplitude of the N1 component was quantified by picking the N1 peak on each participant's average ERP (the most negative local minimum in the time window 25–175 ms postaudible-syllable), as per our previous protocol (Whitford et al., 2017, 2025b). The P2 component was quantified with a time window-based approach (as per our previous protocol) as the average voltage in the time window 162–202 ms postaudible-syllable. For the analysis of the prestimulus activity, the dependent variable was average voltage in the window −500 to 0 ms (i.e., from 500 ms before the audible syllable until the onset of the audible syllable), as per our previously published protocol (Chung et al., 2023). In the case of a main effect of Condition, contrasts were used to unpack the simple effects. If the assumption of sphericity was violated, the Greenhouse–Geisser correction was applied.

For the ERP data, we also performed a Bayesian analysis. The aim of this analysis was to interpret the magnitude of the simple effects in relation to benchmark values rather than to compare competing models [see Kruschke and Liddell (2018) for a discussion of the challenges of model comparison]. By quantifying the probability that a simple effect is as large or larger than a given value, we can evaluate whether there is a non-negligible probability that the observed simple effect has a theoretically meaningful magnitude. We chose this specific study to use as our reference for comparison for two reasons: (1) this study used linked mastoids as the reference, thus making the results more directly comparable to the present study which did the same; (2) it provides a more conservative comparison, since the magnitudes of the observed simple effects were somewhat smaller in Whitford et al. (2025b) than in our original study using this procedure (Whitford et al., 2017). This analysis used the related sample inference routine in SPSS to characterize the posterior distribution for a given simple effect, using diffuse (noninformative) priors for the mean and variance parameters. To calculate the posterior probability of the observed simple effect being greater than the comparison value, we evaluated the complement of the cumulative distribution function of a normal distribution (with mean and variance parameters from the estimated posterior distribution) at the comparison value. We used the relevant simple effect mean from Whitford et al. (2025b) as an indicator of a typical effect size of interest and the simple effect mean divided by two as an indicator of the smallest effect size of interest. To clarify, the Bayesian analysis quantified the probability that the effect magnitudes observed in the present study were as large (or larger) as the typical and smallest effect-size benchmarks based on Whitford et al. (2025b); it did not quantify the probability of the null hypothesis in an absolute sense.

With regard to the time–frequency data, the analysis pipeline was based on the procedure described in Whitford et al. (2025b). The time–frequency data were analyzed with BESA Statistics 2.1 which uses the cluster-based permutation testing algorithm developed by Maris and Oostenveld (2007). Spectral power (µV^2^) for the 40 frequency bands was exported for all 12 central electrodes analyzed in the ERP analysis (viz., Fz, FCz, Cz, CPz, F1, F2, FC1, FC2, C1, C2, CP1, CP2) and compared between the Primed, Misprimed, and Control conditions. Nonparametric permutation testing was performed on the basis of the paired-samples *t* test (two-tailed). The neighbor distance was set to the default setting of 4 cm, the cluster alpha level was set to 0.05, and 10,000 permutations were used. The output is corrected for multiple comparisons, and only those clusters with a cluster value (calculated as the sum of the *t* values of all time–frequency points in the cluster) exceeding 95% of all clusters generated by a random permutation of the data are considered statistically significant.

## Results

**Figure 2.**
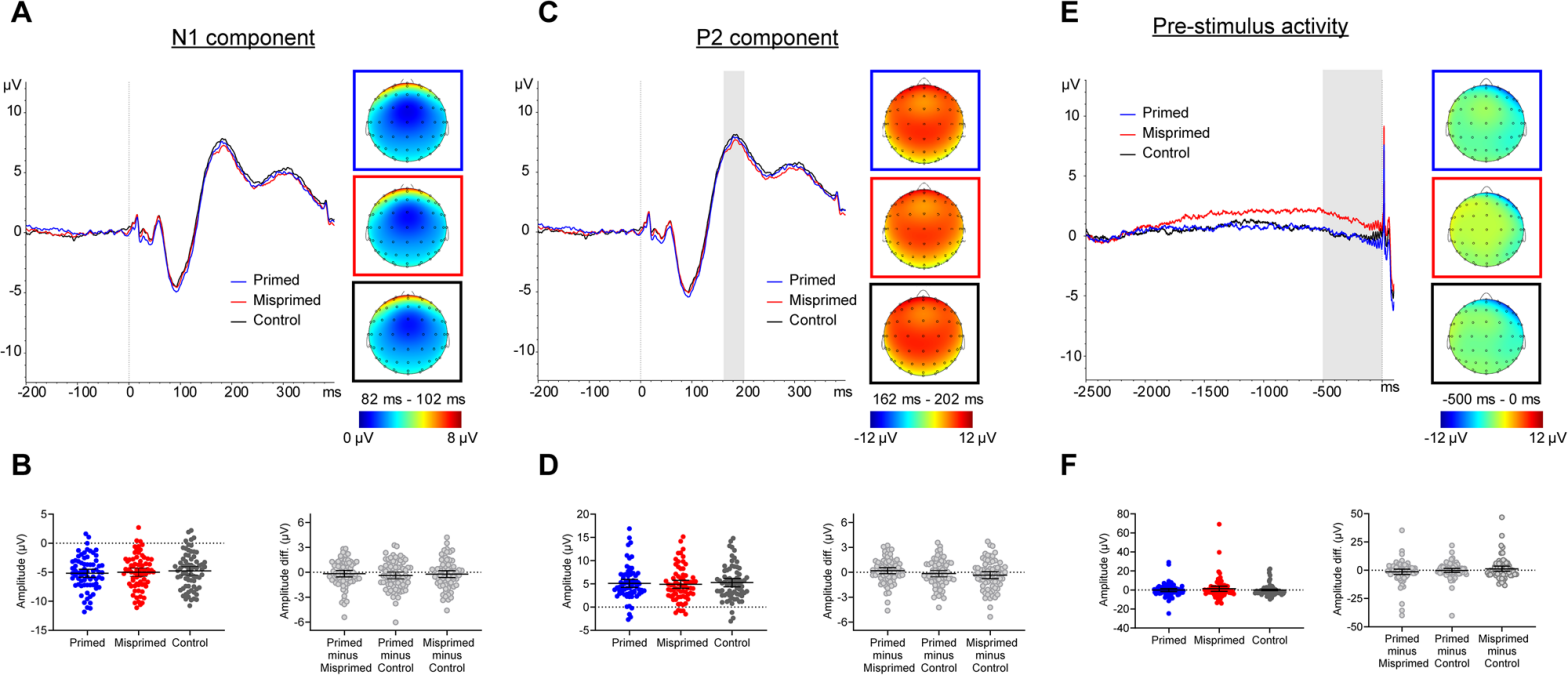
The results of the event-related potential analyses, for the N1, P2, and prestimulus activity. ***A***, The waveforms for the N1 component, for the three conditions (Primed in blue, Misprimed in red, Control in black), time-locked to the onset of the audible syllable. The waveforms are shown collapsed across the nine central electrodes at which N1 was maximal (FCz, FC1, FC2, Cz, C1, C2, Fz, F1, F2). The N1 data were analyzed using a peak-picking approach. ***B***, The scatterplots for the N1 analysis. The left-hand scatterplot depicts the amplitude of the N1 for each condition. Each dot represents a single participant's raw score; the bars represent the mean and 95% CI. The right-hand scatterplot depicts the difference scores on N1 amplitude for the three contrasts-of-interest: namely, Primed minus Misprimed, Primed minus Control, and Misprimed minus Control. ***C***, The waveforms for the P2 analysis, shown collapsed across the nine central electrodes at which P2 was maximal (Cz, C1, C2, FCz, FC1, FC2, CPz, CP1, CP2). The time window for the P2 analysis was 162–202 ms as represented by the gray vertical bar. ***D***, The scatterplots for the P2 component (left-hand scatterplot) and difference scores for the P2 component (right-hand scatterplot). ***E***, The waveforms for the prestimulus activity, shown collapsed across the three central electrodes for which the prestimulus activity was maximal (Cz, FCz, and CPz). The time window for the prestimulus analysis was −500 to 0 ms, as represented by the gray vertical bar. ***F***, The scatterplots for the prestimulus activity, with raw scores on the left-hand scatterplot, and difference scores on the right-hand scatterplot.

Figure 2 shows the auditory-evoked potentials for the three conditions—Primed, Misprimed, and Control—for the N1 component (A,B), the P2 component (C,D), and the prestimulus activity (E,F). These waveforms are time-locked to the onset of the audible syllable and collapsed across electrodes-of-interest as described in Materials and Methods, Statistical analysis.

### N1 component

For N1 amplitude, the repeated-measures ANOVA revealed that the main effect of Condition was not significant (*F*_(2,138)_ = 1.886; *p* = 0.156; $\eta_{p}^{2}=0.027$). Visual inspection of the waveforms provided no indication that N1 amplitude was reduced in the Primed condition relative to the other conditions. Indeed, N1 amplitude was actually numerically greater in the Primed condition (*M* = −5.158) relative to the Control condition (*M* = −4.765). Though this contrast did not reach significance (*t*_(69)_ = 1.92; *p* = 0.06; Cohen's dz = 0.23; 95% CI for difference [−0.803, 0.017]), the mean difference may be suggestive of a small effect of the priming manipulation but in the opposite direction to what we have observed in our previous inner-speech studies. In other words, this trend-level result provides, if anything, evidence against the claim that the inner SIS effect we have previously observed is due to a priming effect. From the posterior distribution (*M* = −0.3931; SD = 0.212), the probability that the current effect was larger than +0.64 µV [i.e., the mean of the Passive vs Match contrast in the Whitford et al. (2025) paper] was *p* < 0.001, while the probability that the current effect was larger than +0.32 µV [i.e., the smallest effect-size-of-interest, defined as half the simple effect mean described in Whitford et al. (2025)] was *p* < 0.001. N1 amplitude also did not differ significantly between the Primed condition (*M* = −5.158) and the Misprimed condition (*M* = 4.984; *t*_(69)_ = 0.892; *p* = 0.375; Cohen's dz = 0.11; 95% CI for difference [−0.563, 0.215]). From the posterior distribution (*M* = −0.1741; SD = 0.200), the probability that the observed effect was larger than +0.39 µV [i.e., the mean of the Match vs Mismatch contrast in the Whitford et al. (2025) paper] was *p* = 0.002, while the probability that the current effect was larger than +0.2 µV [i.e., the smallest effect-size-of-interest, defined as half the simple effect mean described in Whitford et al. (2025)] was *p* = 0.032.

For completeness, there was a significant main effect of Electrode (*F*_(8,552)_ = 100.315; *p* < 0.001; $\eta_{p}^{2}=0.592$), but the Condition * Electrode interaction was not significant (*F*_(16,1104)_ = 1.048; *p* = 0.377; $\eta_{p}^{2}=0.015$).

For the sake of robustness, we also analyzed the N1 data using a time window-based approach. The N1 time window was defined as 82–102 ms, based on the collapsed localizer approach (Luck and Gaspelin, 2017). The pattern of results was identical to the peak-picked data: the main effect of Condition was nonsignificant (*F*_(2,138)_ = 0.572; *p* = 0.560; $\eta_{p}^{2}=0.008$), and none of the pairwise contrasts approached significance (all *p*s > 0.1). For completeness, there was a main effect of Electrode (*F*_(8,552)_ = 90.691; *p* < 0.001; $\eta_{p}^{2}=0.568$), but the Condition * Electrode interaction was nonsignificant (*F*_(16,1104)_ = 0.554; *p* = 0.638; $\eta_{p}^{2}=0.008$).

### P2 component

For the P2 component, repeated-measures ANOVA revealed that the main effect of Condition was not significant (*F*_(2,138)_ = 1.581; *p* = 0.210; $\eta_{p}^{2}=0.022$). The Primed versus Control contrast was nonsignificant (*t*_(69)_ = 0.82; *p* = 0.414; Cohen's dz = 0.10; 95% CI for difference [−0.538, 0.224]), as was the Primed versus Misprimed contrast (*t*_(69)_ = 1.04; *p* = 0.299; Cohen's dz = 0.13; 95% CI for difference [−0.172, 0.553]).

From the posterior distribution (*M* = −0.1569; SD = 0.197), the probability that the observed effect was larger than +0.80 µV [i.e., the mean of the Passive vs Match contrast in the Whitford et al. (2025) paper] was *p* < 0.001, while the probability that the observed effect was larger than +0.40 µV [i.e., the smallest effect-size-of-interest, defined as half the simple effect mean described in Whitford et al. (2025)] was *p* = 0.0024.

For completeness, the main effect of Electrode was significant (*F*_(8,552)_ = 39.020; *p* < 0.001; $\eta_{p}^{2}=0.361$), but the Condition * Electrode interaction was not significant (*F*_(16,1104)_ = 0.917; *p* = 0.428; $\eta_{p}^{2}=0.013$).

### Prestimulus activity

The repeated-measures ANOVA revealed a nonsignificant main effect of Condition (three levels: Primed, Misprimed, Control; *F*_(2,276)_ = 1.012; *p* = 0.359; $\eta_{p}^{2}=0.014$). The Primed versus Control contrast was nonsignificant (*t*_(69)_ = 0.13; *p* = 0.894; Cohen's dz = 0.02; 95% CI for difference [−1.874, 1.640]), as was the Primed versus Misprimed contrast (*t*_(69)_ = 1.15; *p* = 0.255; Cohen's dz = 0.14; 95% CI for difference [−3.797, 1.024]). We also used one-sample *t* tests to investigate whether the average voltage in the prestimulus window (−500 to 0 ms) was significantly different from zero and found that this was not the case for any of the three conditions (Primed, *t*_(69)_ = 0.079; *p* 0.937; Cohen's dz = 0.009; 95% CI for difference [−1.7320, 1.5997]; Misprimed, *t*_(69)_ = 0.967; *p* = 0.337; Cohen's dz = 0.116; 95% CI for difference [−1.4044, 4.0449]; Control, *t*_(69)_ = 0.071; *p* = 0.944; Cohen's dz = 0.008; 95% CI for difference [−1.3951, 1.4974]).

For completeness, we also note that the main effect of Electrode was nonsignificant (*F*_(2,138)_ = 2.514; *p* = 0.103; $\eta_{p}^{2}=0.035$), as was the Condition * Electrode interaction (*F*_(2,138)_ = 2.596; *p* = 0.054; $\eta_{p}^{2}=0.036$).

### Time–frequency analysis

**Figure 3.**
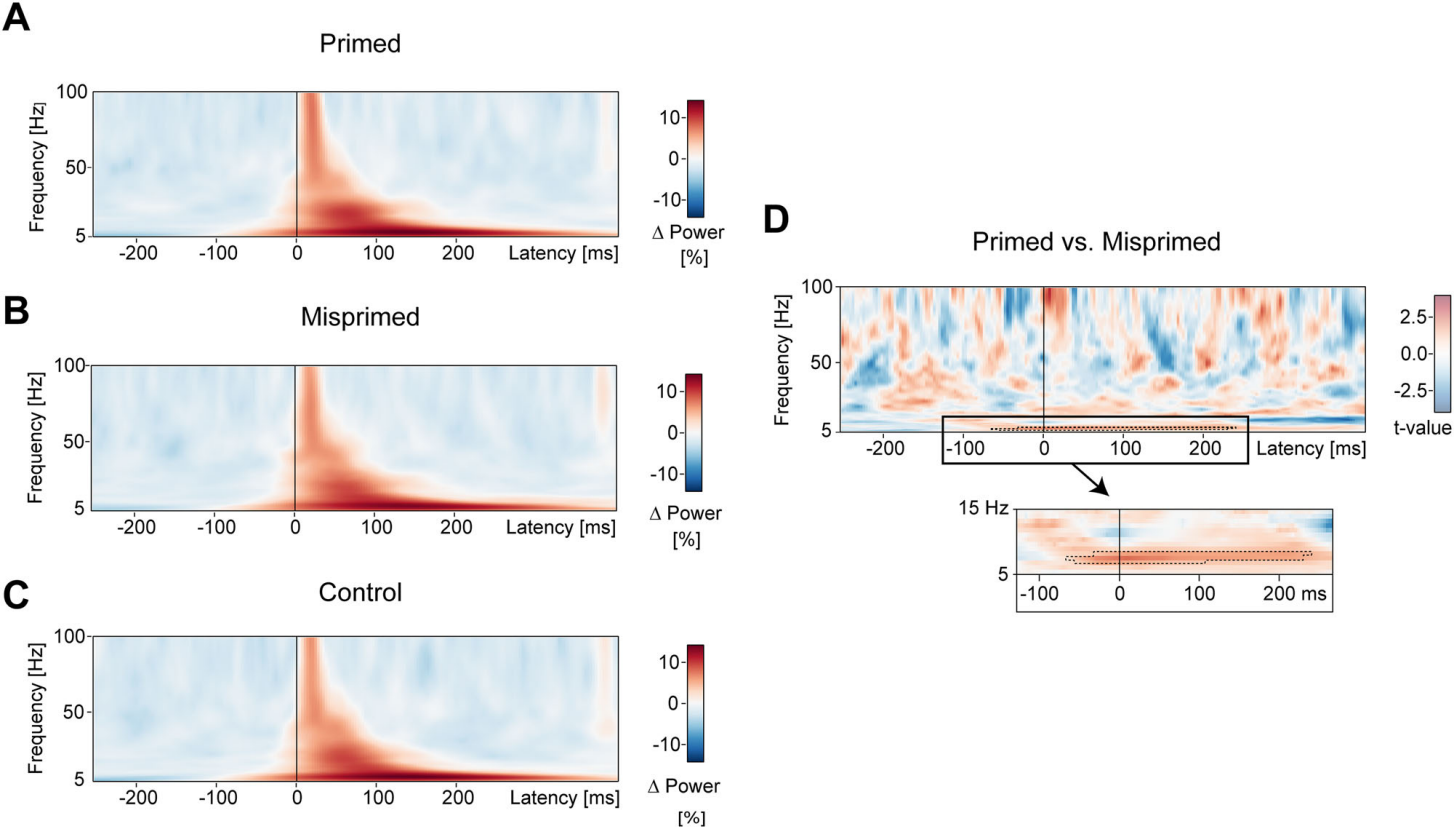
The results of the time–frequency analyses. ***A***, The changes in spectral power from 5–100 Hz across the time window −250 to 400 ms for the Primed condition. ***B***, The corresponding plot for the Misprimed condition. ***C***, The corresponding plot for the Control condition. All time–frequency plots are shown collapsed across the 12 electrodes-of-interest (i.e., Cz, C1, C2, FCz, FC1, FC2, CPz, CP1, CP2, Fz, F1, F2). The time–frequency plots were baseline corrected using a decibel transform and time-locked to the onset of the audible syllable. The units are percentage change in spectral power, relative to the baseline period (−250 to −50 ms). ***D***, The results of the statistical contrasts for the Primed versus Misprimed comparison; the units are *t* values. Nonparametric permutation testing (10,000 permutations) was performed on the basis of a paired-sample *t* test (2-tailed). Only a single cluster was identified which had a cluster value (sum of all *t* values of all pixels in the cluster) that exceeded 95% of all clusters generated by the permutation procedure. This cluster, which is outlined with a dotted line in Panel ***D*** and blown-up in the insert, extended from around −60 to 240 ms and from ∼7–9 Hz and reflected a region of increased power in the Primed condition relative to the Misprimed condition.

Figure 3 shows the spectral power from 5 to 100 Hz for each of the three conditions, namely, Primed (A), Misprimed (B), and Control (C). These time–frequency plots are shown collapsed across the 12 electrodes-of-interest. The time–frequency plots were baseline corrected using a decibel transform and time-locked to the onset of the audible syllable. The units are percentage change in spectral power, relative to the baseline period.

As shown in Figure 3*D*, after controlling for multiple comparisons with a permutation-based cluster analysis, there was a single cluster of pixels that significantly differed in spectral power between the Primed and Misprimed conditions (*t*_max_ = 4.08; *p* = 0.015; latency max = −9 ms; frequency max = 7.3 Hz). This cluster, which extended from around −60 to 240 ms and from around 7–9 Hz, reflected higher power in the Primed condition relative to Misprimed condition. There were no significant clusters when comparing between the Primed and Control conditions or between the Misprimed and Control conditions.

### Behavioral performance

As expected, participants performed at near-ceiling levels for all three conditions when they were asked to report the syllable they were instructed to pay attention to on four randomly selected trials per block. Specifically, participants responded correctly on 99.3% of trials in the Primed condition, 96.8% in the Misprimed condition, and 97.0% in the Control condition.

## Discussion

The aim of this study was to investigate whether the phenomenon of “inner SIS”—as well as the other electrophysiological changes that we have previously observed in our inner-speech task—could be caused by changes in the subjective probability of the (objectively unpredictable) auditory stimuli, as opposed to being caused by inner speech per se. To this end, we designed an experiment based on our established inner-speech task, in which healthy participants (*N* = 70) were not asked to produce inner speech but were instead asked to attend to and remember a syllable presented on the screen at the start of each trial (either /ba/, /bi/ or /fo/). Participants were then presented with one of two audible syllables: /ba/ or /bi/. We compared trials in which the primed syllable either (1) matched the audible syllable that was presented (Primed condition; e.g., prime /ba/, hear /ba/), (2) matched the audible syllable that was not presented (Misprimed condition; e.g., prime /bi/, hear /ba/), or did not match either of the two audible syllables (Control condition; e.g., prime /fo/, hear /ba/). We reasoned that if the changes in ERP and time–frequency activity we have previously observed when participants produced an inner syllable while simultaneously hearing an audible syllable (Whitford et al., 2017; 2025b; Jack et al., 2019; Chung et al., 2023) were actually caused by a change in the subjective probability of hearing the syllable (i.e., as opposed to the inner speech per se), then we should see comparable changes in ERP and time–frequency activity in the current experiment.

To summarize the results: there were no significant between-condition differences on any of the ERP components—N1 amplitude, P2 amplitude, or prestimulus activity. There were also no between-condition differences in gamma-band power (30–100 Hz; though there was a cluster of pixels in the theta/alpha band that differed between the Primed and Misprimed conditions, as discussed below). Furthermore, a Bayesian analysis highlighted the low probability of the magnitude of the observed results being comparable to our previously published data using a version of the task in which participants were required to produce inner speech (Whitford et al., 2025b). Taken together, these results suggest that inner-speech production is a necessary factor for eliciting the above changes in auditory-evoked activity in the context of our typical inner-speech paradigm and that they cannot be explained by changes in the subjective probability of the auditory stimulus.

Earlier we noted that, in a thought-provoking article, Patel (2022) suggested that our previously reported finding of reduced N1 amplitude in the Match condition (relative to the Mismatch condition) of an inner-speech task (Whitford et al., 2017; 2025b; Jack et al., 2019) might not be a consequence of inner speech at all but might instead reflect a expectancy effect in which cueing participants to produce a speech sound primes them to expect that speech sound to occur. The current data argue against this interpretation: when participants were primed with a speech sound in the same way as in our prior studies but were not required to produce inner speech, no between-condition differences in auditory activity were observed. In particular, when participants were primed to hear a certain syllable (i.e., in the Primed condition) but were not asked to produce this syllable in inner speech, this did not result in any reduction in N1 amplitude, relative to when they were primed to hear the other potential audible syllable (i.e., in the Misprimed condition). Moreover, cross-experiment analysis indicated a critical difference between patterns of data observed under “inner-speech” versus “no inner-speech” conditions. Taken together, these results suggest that inner-speech production is a necessary factor for eliciting the observed changes in auditory-evoked activity in the context of our typical inner-speech paradigm, and—contrary to Patel’s (2022) suggestion—these findings cannot be explained by changes in the subjective probability of the auditory stimulus. That is, these results suggest that the N1 suppression effect—or what we have dubbed inner-speaking-induced suppression; “inner SIS”—is instead dependent on the production of inner speech.

As an aside, while participants were not required to produce inner speech in this study, it is possible that they may have subvocally rehearsed the primed syllable even in the absence of explicit inner-speech instructions. It is unlikely that any such rehearsal would influence the N1 elicited by the audible syllable as it would not be tightly time-locked to that syllable (and our previous work suggests that precise time-locking is critical for inner SIS; Jack et al., 2019). Moreover, to the extent that uninstructed inner speech occurred in the current procedure, it would tend to reduce the magnitude of differences in findings between the current study and previous procedures in which participants were required to produce inner speech. Thus, the possibility that participants were subvocally rehearsing the primed syllable in the current task cannot account for our finding of a stark difference in findings between this study and previous research (with regard to patterns of N1 amplitude).

Far from being a purely academic question, establishing the mechanistic basis of inner SIS may have important clinical implications, viz., understanding the etiological basis of psychotic disorders such as schizophrenia. We have recently shown that inner SIS is abnormal in people with schizophrenia-spectrum disorders and that these abnormalities are particularly marked in patients with current auditory-verbal hallucinations [Whitford et al., 2025a; see also Chung et al. (2025) ]. There is a well-established literature indicating that some of the most characteristic symptoms of schizophrenia (e.g., passivity experiences, audible thoughts) are underpinned by abnormalities in distinguishing between “self” and “world” and that these abnormalities may arise from problems in corollary discharges and related mechanisms (Feinberg, 1978; Ford and Mathalon, 2005; Frith, 2005, 2014; Whitford et al., 2012; Pinheiro et al., 2020; Beño-Ruiz-de-la-Sierra et al., 2024a,b). The results of the present study—which suggest that the inner SIS effect is not due to changes in the subjective probability of hearing a sound—leave open the possibility that the inner SIS effect is mediated by corollary discharges. Our hope is that the inner SIS effect could represent a biomarker for corollary-discharge dysfunction in auditory-verbal hallucinations specifically and/or schizophrenia-spectrum disorders more generally.

With regard to the P2 results: a consistent finding in our previous studies using the inner-speech task is that P2 is reduced in the Mismatch condition relative to the Match and Passive conditions, typically with a large effect size (Whitford et al., 2025b). In keeping with the suggestion that the P2 reflects “the conscious evaluation of action outcomes” (Crowley and Colrain, 2004; Stekelenburg and Vroomen, 2012; Knolle et al., 2019; Korka et al., 2022; Whitford et al., 2025b), we have previously suggested that the P2 amplitude reduction in the Mismatch condition might reflect participants “consciously recognizing the difference in content between the inner and audible syllables” (Whitford et al., 2025b) or, in other words, a cognitive expectancy violation. We have also suggested that a similar mechanism might underlie the reduced gamma-band activity we have previously observed in the Mismatch condition, relative to Match, in the vicinity of the P2 window (Whitford et al., 2025b). In the present study, we did not observe any between-condition differences in P2 amplitude, which suggests that simply recognizing the difference between a primed syllable and an audible syllable is not sufficient to induce changes in the P2 component. This finding is consistent with the idea that there is something functionally important about the active process of producing inner speech with regard to generating the changes in P2 amplitude that we have previously observed in our inner-speech task.

To summarize the Prestimulus results: in the present study, none of the three conditions differed either from each other, or from zero, in the prestimulus time window (i.e., from 500 ms prestimulus to stimulus onset). This result is instructively different from the findings of Chung et al. (Chung et al., 2023), who used our typical inner-speech task design. Chung et al. found evidence of a slow, negative-going potential in all three conditions their study; i.e., both in the two inner-speech conditions (Match and Mismatch) but also in the Passive condition in which participants were not required to produce inner speech. We have previously suggested that this slow, negative-going potential in the Passive condition may actually reflect a contingent negative variation (CNV) elicited by the imperative aspect of the experimental paradigm (“ready … set … act!”; Teodoro et al., 2020). If this is the case, then one possible interpretation of the fact that we did not observe a CNV in either condition is that the mental action of “paying attention to a syllable” was either not as willful as the production of inner speech or not as tightly time-locked to the sound-time, or both, and thus did not contain the imperative aspect necessary to elicit a CNV.

[As an aside, it is notable that in Fig. 2*E* the Prestimulus waveform appeared somewhat higher (i.e., more positive) for the Misprimed condition compared with both the Primed and Control waveforms. This apparent difference was driven by two participants (visible as outliers in Fig. 2*F*) who showed an unusual pattern of prestimulus activity; specifically, these participants showed a large, positive-going waveform that continued to elevate throughout the prestimulus period. It should be emphasized that this between-condition difference in CNV did not approach statistical significance with these participants included (*p* = 0.359). Moreover, we verified that excluding data from these two outlying participants did not change the pattern of significant and nonsignificant findings in any of the reported analyses.]

As noted above, the current study was designed to test Patel’s (2022) suggestion that our prior findings of “inner SIS” (Whitford et al., 2017; Jack et al., 2019; Whitford et al., 2025b) may reflect priming of participants' subjective belief as to which sound they would hear. It is important to note that in this prior work, the “prime” actually provided no information about which audible syllable would occur: for example, given the prime /ba/, the audible syllable was equally likely to be /ba/ or /bi/. As such, Patel's hypothesis rests on the ability of the prime to produce a subjective expectation of an upcoming syllable even in the absence of any objective basis for forming that expectation. To test this hypothesis, in the current study, we primed participants in the same way as in our prior inner-speech studies—i.e., the prime provided no objective information regarding the upcoming sound—the only difference being that we did not ask participants to produce inner speech at the sound time. Under these conditions, we found no impact of the prime on auditory activity, showing that priming-induced subjective expectancy was not the source of the “inner SIS” effect observed in our prior inner-speech studies. That said, it remains an open question as to whether there are conditions under which expectancy alone can modulate auditory processing. For example, it is possible that expectancy could induce a change in processing of the audible syllable if (unlike in the current study) the prime provided objective, predictive information that allowed for a valid expectancy to be formed, e.g., if the prime /ba/ was always (and only) followed by the sound /ba/. While this is clearly a very different situation from the “unpredictable environment” (Patel, 2022) characteristic of our typical inner-speech task (in which the occurrence of a specific audible syllable is unpredictable from trial-to-trial), it is nevertheless a worthwhile topic for future study.

The only between-condition difference we observed in the present study was of a single cluster of pixels in the theta/alpha band that showed reduced power in the Misprimed condition relative to the Primed condition (Fig. 3*D*). This cluster had a relatively narrow frequency band (∼7–9 Hz) but a wide temporal extent (around −60 to +240 ms). Notably, this cluster was similar in shape—in terms of both its frequency range and temporal extent—to one of the clusters we identified in our previous study [as shown in Fig. *4*B of Whitford et al. (2025b)] which showed reduced power in the Mismatch condition relative to the Match condition (consistent with the results of the present study). This result has two important implications. Firstly, it suggests that the reduced power we observed in this specific cluster in our previous study (Whitford et al., 2025b) may have been driven by a “priming-like” effect, which is consistent with the prediction of Patel (2022). Secondly, the fact that we observed a significant between-condition difference in time–frequency activity in the present study provides further evidence that participants were paying attention to the task (in addition to the behavioral data). Our reasoning is that if participants were not paying attention to the task, then there would be no distinction between Primed and Misprimed trials, and thus no difference in evoked activity would be observed. In other words, our identification of a between-condition difference in time–frequency activity represents a “manipulation check” of the priming manipulation and provides evidence against the possibility that the otherwise null results we observed were simply due to participants failing to engage in the task. However, it should be noted that this analysis was exploratory. As such, the interpretations discussed above, while plausible, remain tentative and require replication in future research before firm conclusions can be drawn.

A notable feature of the experimental design is that the blocks were organized by trial type; that is, participants completed 24 “Prime /ba/” trials, for example, followed by 24 “Prime /bi/” trials, etc. We used this “blocked” approach for consistency with the previous studies on which the current study is based. However, a potential limitation of this approach is that it opens up the possibility of habituation/adaptation effects developing across the course of the block. An alternative approach would have been to change the trial-type every trial. While such a “trial-by-trial” approach would have avoided the possibility of habituation/adaptation across the block, it could potentially have led to other challenges, such as difficulty maintaining concentration to ever-changing task instructions (and would have created a confound when attempting to compare results with previous findings). Nevertheless, replicating these results using a trial-by-trial design would be a worthwhile aim for future research.

A limitation of contrasting the absence of between-condition ERP effects in the present study against the presence of between-condition ERP effects in our previous inner-speech studies is that participants did not actually complete our typical inner-speech task. In other words, it is possible that, for some reason, our participants would not have shown the between-condition differences in N1, P2, prestimulus activity, and oscillatory activity that we have previously observed with our inner-speech task. We think this possibility is unlikely given that we have now replicated these effects in five independent samples of healthy participants (Whitford et al., 2017; 2025b; Jack et al., 2019; Chung et al., 2023, 2025; Berryman et al., 2025). Nevertheless, it would have been ideal if our participants had completed both the typical task (which involved inner speech) and the modified task (which did not) and showed between-condition differences in the former task but not the latter, though this would have likely required multiple testing sessions per participant given the lengthy duration of the experimental tasks (at ∼45 min each). Another limitation is that while we did ask participants to pay attention to and remember which syllable was presented as the prime, we did not explicitly ask participants to rate their subjective expectation of what syllable they expected to hear on the next trial. While the fact that participants' accuracy was near-ceiling on the behavioral task provides evidence that they were able to remember the primed syllable, this does not necessarily imply that the visual cue affected their subjective expectation of actually hearing the primed syllable. Finally, while our task attempted to manipulate participants' subjective expectation of hearing a syllable (i.e., without changing its objective probability), it is possible that the cognitive demands of the task differed from those associated with producing inner speech in our typical protocol. It is possible (though, we suspect, unlikely) that such differences in cognitive load could underlie the present study's failure to observe between-condition differences in N1, P2, prestimulus activity, and gamma-band power. Testing this possibility would be a worthwhile aim for future research, while acknowledging that quantifying the cognitive load associated with producing an inner syllable is a challenging endeavor.

In conclusion, in the present study, priming participants to hear a specific audible syllable did not reproduce the EEG changes that we have previously shown to be associated with inner-speech production (i.e., changes in N1, P2, prestimulus activity, and gamma-band power). This result suggests that a simple, priming-based effect is unlikely to be sufficient to account for these EEG changes; they seem to require the active production of inner speech. Future work on the phenomenon of “inner speaking-induced suppression” could explore the effect of changing participants’ subjective expectations of hearing an audible syllable by manipulating their objective probability of occurrence.

## Synthesis

Reviewing Editor: Anne Keitel, University of Dundee Decisions are customarily a result of the Reviewing Editor and the peer reviewers coming together and discussing their recommendations until a consensus is reached. When revisions are invited, a fact-based synthesis statement explaining their decision and outlining what is needed to prepare a revision will be listed below. The following reviewer(s) agreed to reveal their identity: Ana Pinheiro.

The manuscript "Exploring the mechanisms underlying the phenomenon of 'Inner Speaking-Induced Suppression" has been reviewed by two expert reviewers and the editor. The reviewers found the study well-motivated and methodologically sound, praising its clear conceptual rationale, strong sample size, and novel use of a cueing paradigm with EEG to test a theoretically important alternative explanation of inner speech-induced suppression. However, the reviewers thought the conclusions were overstated, noting that the null results rule out only a simple inattention account rather than expectancy more broadly, and that the lack of direct expectancy measures, within-subject comparisons, and clearer statistical justification for null findings significantly weakened the central mechanistic claims. The decision was therefore "revise and re-review". You find the summarised comments below, please respond to each in a point-by-point manner.

Overall assessments - no need to respond to these

(1) This manuscript addresses an important and timely question regarding the mechanisms underlying speech-induced suppression (SIS) during inner speech. Specifically, the authors test whether the inner SIS effect can be explained by expectancy-based processes, as opposed to corollary discharge mechanisms. The study is well-motivated, methodologically sound, and clearly reported. The use of a cueing paradigm combined with EEG provides a novel and appropriate approach to disentangling these competing accounts. The finding of no expectancy effect is interesting and potentially important for theoretical models of inner speech. Overall, the manuscript is strong, but I have a few comments and questions that I believe would help clarify the interpretation of the findings and strengthen the conclusions.

(2) Advances the field: This manuscript provides a useful, though relatively circumscribed, advance in the field by testing an important alternative explanation of previously reported inner speaking-induced suppression (inner SIS), namely that the effect reflects expectancy or priming rather than inner speech production itself. Using a modified version of the paradigm, the authors show that simply attending to and remembering a prime syllable is not sufficient to reproduce the ERP effects previously associated with inner speech. This helps constrain the interpretation of earlier findings and argues against a simple inattention or cue-memory account. At the same time, the advance is more incremental than definitive, because the present design does not directly measure subjective expectancy or fully isolate it from covert rehearsal or other internal strategies. Overall, the manuscript advances the field by refining the boundary conditions of the phenomenon, even if it does not yet conclusively identify its mechanism.

Overall assessment: This manuscript addresses a clear and theoretically important question: whether previously reported "inner speaking-induced suppression" (inner SIS) reflects a corollary-discharge-like mechanism linked to inner speech production, or instead a more general expectancy/priming effect. The study is well motivated, the logic of the control manipulation is straightforward, and the null ERP findings are potentially informative because they bear directly on the interpretation of an emerging literature. The authors also go beyond simple null-hypothesis testing by comparing the present effects to prior inner-speech results using a Bayesian framing. The core design is easy to understand: participants were primed with a syllable but did not generate inner speech, allowing the authors to test whether priming alone reproduces earlier ERP effects attributed to inner speech.

At the same time, the manuscript currently overstates what can be concluded from these data. The absence of condition differences in this expectancy-only task does not, on its own, establish that corollary discharge is the operative mechanism, nor does it fully exclude alternative expectancy-based accounts. The behavioural checks convincingly show that participants attended to and remembered the prime, which is an important strength of the design. However, these measures do not directly assess whether participants formed a trial-wise expectation that the matching sound was more likely, nor do they clarify the covert strategy used to maintain the prime. As such, the study strongly rules out a simple inattention account, but less decisively isolates subjective expectancy per se. Relatedly, the design also does not fully rule out the possibility that some participants engaged in covert rehearsal (inner-speech-like) strategies when maintaining the cue. The manuscript acknowledges another important limitation; namely, that the same participants did not complete both the canonical inner-speech task and the modified task; but in my view this limitation is central rather than peripheral to the strength of the inference.

Overall, this is a thoughtful and potentially valuable contribution, but the conclusions would benefit from a more cautious interpretation and from clarification of several methodological and analytical points.

Main strengths: The manuscript has a strong conceptual rationale. It directly addresses an explicit alternative interpretation from Patel (2022), rather than merely extending prior findings. That adversarial framing is a real strength.

The design is clean and easy to follow. The three conditions (Primed, Misprimed, and Control) are sensible for separating content match, content mismatch, and a neutral prime that is never heard. The figure and task description make the paradigm unusually transparent.

The sample size is good for an EEG study of this kind (final N = 70), and the authors report artefact exclusions and usable trial counts per condition. That improves confidence that the null results are not trivially due to poor data retention.

The manuscript tests several previously reported signatures from the authors' prior inner-speech work (N1, P2, pre-stimulus activity, gamma-band power, and broader time-frequency effects) rather than focusing selectively on a single endpoint.

Reviewer comments - please respond to these

Conceptual

1. The central inferential leap is too strong. The study shows that this specific expectancy-only manipulation did not recapitulate prior inner-speech effects. That supports the claim that simple cue-based priming, under these task conditions, is insufficient. It does not yet justify the stronger claim that the effect is therefore "more likely driven by corollary discharge." The discussion partly acknowledges this, but the abstract, significance statement, and conclusion remain too definitive. Please temper the causal/mechanistic language throughout. I would revise statements such as "inner SIS is more likely driven by corollary-discharge-related mechanisms" to something closer to: "The present data indicate that a simple cue-based priming manipulation, in the absence of instructed inner speech, is insufficient to reproduce previously reported inner-SIS effects." That claim is fully supported. The stronger mechanistic conclusion is not. This issue appears in the Abstract, Significance Statement, Discussion, and final Conclusion.

2. The manipulation is framed as "expectancy-only," but the paper does not directly measure expectancy. Participants were instructed to attend to and remember the syllable, and accuracy was near ceiling on the memory probes, which confirms compliance with remembering the cue, not necessarily that they formed a strong auditory expectation or that this expectation matched the state Patel hypothesised. Without trial-wise expectancy ratings, confidence judgments, or a separate behavioural signature of anticipation, the study does not fully validate the targeted construct.

3. Although the authors argue that participants were engaged with the task (based on behavioral performance and time-frequency results), the cognitive demands of attending to and maintaining a syllable differ from those of actively generating inner speech. It would be useful to discuss whether the absence of effects could reflect differences in information processing or task engagement, rather than the absence of expectancy per se.

4. The comparison with previous inner-speech datasets is informative but limited by the between-sample design. The authors themselves note that the same participants did not perform both tasks. This matters because the main argumentative force of the paper rests on the contrast between "inner speech present" and "inner speech absent." A within-subject comparison would have been more decisive. As written, the paper should present the present study as a strong constraint on one alternative account, not as a near-resolution of mechanism.

5. Clarify what exactly was manipulated: priming, attention, working-memory maintenance, or expectancy? Participants were explicitly instructed to attend to and remember the visual syllable, and this was checked behaviourally. That creates a state involving attentional maintenance and mnemonic set, but not necessarily auditory prediction. Please discuss more carefully why this manipulation should instantiate Patel's proposed subjective expectancy account, and where it may fail to do so.

6. Address the possibility of spontaneous covert rehearsal. Because participants were shown BA/BI/FO and asked to remember it, some may have sub-vocally rehearsed it despite the absence of explicit inner-speech instructions. If so, the condition is not strictly "inner speech absent." This possibility cuts both ways: it may dilute the manipulation logic, but it also makes the null result more interesting. Either way, it should be discussed directly.

7. The discussion of CNV/pre-stimulus activity is interesting but speculative. The authors suggest that absence of pre-stimulus effects here may reflect the reduced "imperative" quality of attending to a syllable relative to generating inner speech. That is plausible, but it should be framed as one possible interpretation rather than a likely explanation.

8. Consider whether the Control condition is psychologically neutral. /fo/ is not one of the audible alternatives, which is useful experimentally, but it may differ from /ba/ and /bi/ in phonological familiarity, rehearsal strategy, or categorical relation to the upcoming sound set. A brief justification of this choice would help. Why /fo/ specifically? Could a non-speech visual cue have been an equally valid control?

9. Improve the logic of the main conclusion. I would recommend restructuring the final paragraph around three claims: first, this expectancy-only manipulation did not reproduce prior ERP findings; second, therefore simple cue-maintenance/priming is unlikely to be sufficient; third, future work should compare expectancy manipulations with objective predictive validity and a within-subject inner-speech manipulation. That would make the paper stronger and more precise.

Methodological

10. The statistical treatment of null effects needs clearer justification. Repeated-measures ANOVAs yielding non-significant main effects do not, by themselves, establish evidence for absence. The Bayesian comparison to prior effect sizes is a useful addition, but the manuscript should explain that this analysis addresses whether the present effects are likely to be as large as previously reported effects, not whether the null is true in an absolute sense. The current wording may encourage over-interpretation.

11. Related, make the inferential target of the Bayesian analysis more explicit. The manuscript compares present simple effects to previously observed values and reports the posterior probability that the present effect exceeds those prior benchmarks. This is not the same as a Bayes factor for H0 versus H1, nor a direct estimate that the effect is absent. Please explain more clearly what this analysis does and does not show, and consider reporting interval estimates around present condition differences as well.

12. Further related, because the main conclusions rely on null effects, it would be valuable to provide a more explicit assessment of statistical sensitivity. For instance, reporting the smallest effect size of interest, equivalence testing, or Bayes factors specifically quantifying evidence for the null hypothesis would help readers evaluate whether the study was sufficiently powered to detect theoretically meaningful effects.

13. Report effect sizes and uncertainty for the key pairwise contrasts in the main text. The manuscript reports means and p values for some contrasts, but it would help readers to see effect sizes and confidence intervals or credible intervals for Primed-Misprimed and Primed-Control differences for N1, P2, and pre-stimulus activity. Since the paper hinges on null or near-null effects, interval estimates are especially important.

14. The near-significant Primed vs Control N1 difference deserves brief treatment. The N1 in the Primed condition was numerically more negative than in Control, with p = .06. I do not think this undermines the main conclusion, but it should be discussed because it runs opposite to the expected suppression direction and may indicate small unspecific effects of the task manipulation.

15. Similarly, clarify the rationale for the N1 quantification approach. N1 was quantified via peak-picking within 25-175 ms, whereas P2 used a windowed mean. Peak measures can be noisier and more sensitive to latency jitter. Please explain why peak-picking remains the preferred choice here, and consider adding a supplementary mean-amplitude analysis for N1 over a predefined window to show robustness.

16. Expand the interpretation of the 7-9 Hz cluster or temper it. At present, the theta/alpha cluster is used both as evidence for a priming-like effect and as a manipulation check. Both are plausible, but the current evidence is thin. The manuscript should either present this as exploratory or provide stronger grounding for why that frequency range and temporal extent should index priming/expectancy.

17. Please discuss block structure as a possible source of sustained-state effects. Blocks were organised by prime type ("Prime /ba/," "Prime /bi/," "Prime /fo/"). This may induce sustained context or habituation/adaptation effects beyond trial-wise priming. This concern could be addressed in future work by using randomised trial-by-trial cues and explicitly manipulating cue validity. I do not see this as fatal, but it should be acknowledged, particularly since the theoretical question concerns subjective expectation.

18. Regarding Figure 2, Panel E appears to suggest potential differences between conditions that are not reflected in the reported statistical results. The authors may wish to verify that the plotted data and exported values are consistent, and clarify whether any visualization choices (e.g., scaling or averaging procedures) could account for this apparent discrepancy.

## Author Response

eNeuro - Reponse to Reviewers We thank the Reviewers for their extremely helpful comments on the manuscript. Please find our responses below, which we have addressed on a point-by-point basis. The reviewer comments have been pasted in full in bold text, and our responses are in regular text. Any additions or changes to the revised manuscript are presented in blue font.

Reviewer comments - please respond to these Conceptual 1. The central inferential leap is too strong. The study shows that this specific expectancy-only manipulation did not recapitulate prior inner-speech effects. That supports the claim that simple cue-based priming, under these task conditions, is insufficient. It does not yet justify the stronger claim that the effect is therefore "more likely driven by corollary discharge." The discussion partly acknowledges this, but the abstract, significance statement, and conclusion remain too definitive. Please temper the causal/mechanistic language throughout. I would revise statements such as "inner SIS is more likely driven by corollary-discharge-related mechanisms" to something closer to: "The present data indicate that a simple cue-based priming manipulation, in the absence of instructed inner speech, is insufficient to reproduce previously reported inner-SIS effects." That claim is fully supported. The stronger mechanistic conclusion is not. This issue appears in the Abstract, Significance Statement, Discussion, and final Conclusion.

We have toned-down the causal/mechanistic language throughout, and have made the following changes to the Abstract, Significance Statement, and Discussion (including a change to the concluding sentence): (Abstract). The present data indicate that a simple cue-based priming manipulation, in the absence of inner speech, is insufficient to reproduce previously reported inner-SIS effects. (Significance Statement). This result suggests that a priming manipulation, in the absence of inner speech, is insufficient to reproduce previously reported inner-SIS effects. (Discussion). The results of the present study - which suggest that the inner SIS effect is not due to changes in the subjective probability of hearing a sound - leave open the possibility that the inner SIS effect is mediated by corollary discharges. Our hope is that the inner SIS effect could represent a biomarker for corollary-discharge dysfunction in auditory-verbal hallucinations specifically and/or schizophrenia-spectrum disorders more generally. (Conclusion). In conclusion, in the present study, priming participants to hear a specific audible syllable did not reproduce the EEG changes that we have previously shown to be associated with inner speech production (i.e., changes in N1, P2, pre-stimulus activity, and gamma-band power). This result suggests that a simple, priming-based effect is unlikely to be sufficient to account for these EEG changes; they seem to require the active production of inner speech. Future work on the phenomenon of 'inner speaking-induced suppression' could explore the effect of changing participants' subjective expectations of hearing an audible syllable by manipulating their objective probability of occurrence.

2. The manipulation is framed as "expectancy-only," but the paper does not directly measure expectancy. Participants were instructed to attend to and remember the syllable, and accuracy was near ceiling on the memory probes, which confirms compliance with remembering the cue, not necessarily that they formed a strong auditory expectation or that this expectation matched the state Patel hypothesised. Without trial-wise expectancy ratings, confidence judgments, or a separate behavioural signature of anticipation, the study does not fully validate the targeted construct.

We agree that it might have been a good idea to explicitly ask participants to rate their subjective expectation of what syllable they expected to hear on the next trial. We now acknowledge this point in the Limitations section of the revised manuscript.

Another limitation is that while we did ask participants to pay attention to and remember which syllable was presented as the prime, we did not explicitly ask participants to rate their subjective expectation of what syllable they expected to hear on the next trial. While the fact that participants' accuracy was near-ceiling on the behavioural task provides evidence that they were able to remember the primed syllable, this does not necessarily imply that the visual cue affected their subjective expectation of actually hearing the primed syllable.

3. Although the authors argue that participants were engaged with the task (based on behavioral performance and time-frequency results), the cognitive demands of attending to and maintaining a syllable differ from those of actively generating inner speech. It would be useful to discuss whether the absence of effects could reflect differences in information processing or task engagement, rather than the absence of expectancy per se.

We have added the following section to the Limitations section of the Discussion.

Finally, whilst our task attempted to manipulate participants' subjective expectation of hearing a syllable (i.e., without changing its objective probability), it is possible that the cognitive demands of the task differed from those associated with producing inner speech in our typical protocol. It is possible (though, we suspect, unlikely) that such differences in cognitive load could underlie the present study's failure to observe between-condition differences in N1, P2, pre-stimulus activity and gamma-band power. Testing this possibility would be a worthwhile aim for future research, while acknowledging that quantifying the cognitive load associated with producing an inner syllable is a challenging endeavour.

4. The comparison with previous inner-speech datasets is informative but limited by the between-sample design. The authors themselves note that the same participants did not perform both tasks. This matters because the main argumentative force of the paper rests on the contrast between "inner speech present" and "inner speech absent." A within-subject comparison would have been more decisive. As written, the paper should present the present study as a strong constraint on one alternative account, not as a near-resolution of mechanism.

We have acknowledged in the manuscript that the study would have been improved if participants had also performed the 'traditional' paradigm (see Limitations section in the original manuscript). However, as we also note in the manuscript, running both tasks would not have been feasible in a single experimental session (given the lengthy duration of the task at around 45 minutes), and thus would have required testing over multiple sessions, which adds a layer of complexity (e.g., regarding task order effects, differences in participant state between testing sessions, etc.). Furthermore, we have little reason to believe that the participants would not have shown the standard effects, given that we have now replicated these effects numerous times in independent samples. Specifically, we have now replicated the N1 and P2 results in four published papers (including the Whitford et al., (2025) eNeuro study, which had n = 128 participants), and the pre-stimulus results in two published papers (Chung et al., (2025) and Chung et al., (2023)). Regardless, we have attempted to tone down our language regarding the mechanistic implications of the findings, as detailed in our responses to Point 1.

5. Clarify what exactly was manipulated: priming, attention, working-memory maintenance, or expectancy? Participants were explicitly instructed to attend to and remember the visual syllable, and this was checked behaviourally. That creates a state involving attentional maintenance and mnemonic set, but not necessarily auditory prediction. Please discuss more carefully why this manipulation should instantiate Patel's proposed subjective expectancy account, and where it may fail to do so.

We attempted to manipulate participants' subjective expectation of hearing the cued syllable by instructing them to attend-to-and-remember-it prior to the onset of each trial (which they did successfully, based on the near-ceiling levels of accuracy in the behavioural task). This manipulation was based on Patel's hypothesis that priming participants "to expect that speech sound to occur [will result] in the attenuation of the speech sound that matched the cue-based expectation". Manipulating the subjective expectancy of hearing a syllable without changing its objective probability is clearly a challenging endeavour, and it is difficult to quantify how successful our manipulation was (see also our response to Point 2). A different, more direct, approach would have been to explicitly instruct participants to change their subjective probability (e.g., "On this trial, you should expect that the syllable /ba/ will be played through your headphones"). However, in this case, participants would quickly realize (i.e., within a couple of trials) that they were being deceived (i.e., as the objective probability of hearing /ba/ was always 50%), which could have confounded the results and complicated their interpretation. Given this, we feel as though our attempt to manipulate participants' subjective expectation of hearing a syllable without changing its objective probability was reasonable.

6. Address the possibility of spontaneous covert rehearsal. Because participants were shown BA/BI/FO and asked to remember it, some may have sub-vocally rehearsed it despite the absence of explicit inner-speech instructions. If so, the condition is not strictly "inner speech absent." This possibility cuts both ways: it may dilute the manipulation logic, but it also makes the null result more interesting. Either way, it should be discussed directly.

In a purely mental task such as this (i.e., given the absence of any overt responses), it is possible that participants engaged in covert inner speech, e.g., by sub-vocally rehearsing the syllable that they were being instructed to remember. In this case, it would not be accurate to call the primed conditions 'inner speech absent'. However, even if participants were engaging in sub-vocal rehearsal, we suggest that it is probably unlikely that this rehearsal was tightly time-locked to the audible syllable, as if it was then we would expect to see the changes in N1, P2, pre-stimulus activity and gamma-band activity that we observed in our previous studies, but which we did not.

As suggested by the reviewers, if participants were producing inner speech in this task, then this would (if anything) be expected to increase the difference between the Primed and Misprimed conditions. We have now added a discussion of this issue to the revised manuscript, as follows: (As an aside, while participants were not required to produce inner speech in this study, it is possible that they may have sub-vocally rehearsed the primed syllable even in the absence of explicit inner-speech instructions. It is unlikely that any such rehearsal would influence the N1 elicited by the audible syllable as it would not be tightly time-locked to that syllable (and our previous work suggests that precise time-locking is critical for inner-SIS: Jack et al. 2017). Moreover, to the extent that uninstructed inner speech occurred in the current procedure, it would tend to reduce the magnitude of differences in findings between the current study and previous procedures in which participants were required to produce inner speech. Thus, the possibility that participants were sub-vocally rehearsing the primed syllable in the current task cannot account for our finding of a stark difference in findings between this study and previous research (with regard to patterns of N1 amplitude).

7. The discussion of CNV/pre-stimulus activity is interesting but speculative. The authors suggest that absence of pre-stimulus effects here may reflect the reduced "imperative" quality of attending to a syllable relative to generating inner speech. That is plausible, but it should be framed as one possible interpretation rather than a likely explanation.

We have adjusted our language in the relevant section, which now reads as follows:

If this is the case, then one possible interpretation of the fact that we did not observe a CNV in either condition is that the mental action of 'paying attention to a syllable' was either not as wilful as the production of inner speech, or not as tightly time-locked to the sound-time, or both, and thus did not contain the imperative aspect necessary to elicit a CNV.

8. Consider whether the Control condition is psychologically neutral. /fo/ is not one of the audible alternatives, which is useful experimentally, but it may differ from /ba/ and /bi/ in phonological familiarity, rehearsal strategy, or categorical relation to the upcoming sound set. A brief justification of this choice would help. Why /fo/ specifically? Could a non-speech visual cue have been an equally valid control? We chose the syllable /fo/ for the control condition simply because it had similar properties to the other two syllables used in the task (/ba/ and /bi) in that it was a single-syllable, two-letter sound which is straightforward to pronounce and which is commonly used in English. We now make this point explicit in the revised manuscript:

We chose the syllable /fo/ for the Control condition simply because it had similar properties to the two other syllables used in the task (/ba/ and /bi/); that is, it is a one-syllable, two-letter sound which is straightforward to pronounce and commonly used in English.

We think that the similarities between the syllables probably meant that /fo/ was a better control stimulus than a non-speech visual cue, given that it is not clear how a non-speech visual cue would have affected the auditory-evoked potential (though we acknowledge that it may not have made any difference).

9. Improve the logic of the main conclusion. I would recommend restructuring the final paragraph around three claims: first, this expectancy-only manipulation did not reproduce prior ERP findings; second, therefore simple cue-maintenance/priming is unlikely to be sufficient; third, future work should compare expectancy manipulations with objective predictive validity and a within-subject inner-speech manipulation. That would make the paper stronger and more precise.

We have restructured the final paragraph as per the reviewers' suggestion. The revised paragraph now reads as follows:

In conclusion, in the present study, priming participants to hear a specific audible syllable did not reproduce the EEG changes that we have previously shown to be associated with inner speech production (i.e., changes in N1, P2, pre-stimulus activity, and gamma-band power). This result suggests that a simple, priming-based effect is unlikely to be sufficient to account for these EEG changes; they seem to require the active production of inner speech. Future work on the phenomenon of 'inner speaking-induced suppression' could explore the effect of changing participants' subjective expectations of hearing an audible syllable by manipulating their objective probability of occurrence.

Methodological 10. The statistical treatment of null effects needs clearer justification. Repeated-measures ANOVAs yielding non-significant main effects do not, by themselves, establish evidence for absence. The Bayesian comparison to prior effect sizes is a useful addition, but the manuscript should explain that this analysis addresses whether the present effects are likely to be as large as previously reported effects, not whether the null is true in an absolute sense. The current wording may encourage over-interpretation.

This is an important point to clarify, and accordingly we have added the following sentence to the Statistical Analysis section of the revised manuscript:

To clarify, the Bayesian analysis quantified the probability that the effect magnitudes observed in the present study were as large (or larger) as the typical and smallest effect-size benchmarks based on Whitford et al. (2025); it did not quantify the probability of the null hypothesis in an absolute sense.

11. Related, make the inferential target of the Bayesian analysis more explicit. The manuscript compares present simple effects to previously observed values and reports the posterior probability that the present effect exceeds those prior benchmarks. This is not the same as a Bayes factor for H0 versus H1, nor a direct estimate that the effect is absent. Please explain more clearly what this analysis does and does not show, and consider reporting interval estimates around present condition differences as well.

We have added the following to the methods to clarify our Bayesian analysis approach:

For the ERP data, we also performed a Bayesian analysis. The aim of this analysis was to interpret the magnitude of the simple effects in relation to benchmark values, rather than to compare competing models (see Kruschke &Liddell, 2018, for a discussion of the challenges of model comparison). By quantifying the probability that a simple effect is as large or larger than a given value, we can evaluate whether there is a non-negligible probability that the observed simple effect has a theoretically meaningful magnitude.

With regards to the interval estimates: as per the reviewers' request, we have now added 95% CI ranges around the condition differences, as outlined in Point 13.

12. Further related, because the main conclusions rely on null effects, it would be valuable to provide a more explicit assessment of statistical sensitivity. For instance, reporting the smallest effect size of interest, equivalence testing, or Bayes factors specifically quantifying evidence for the null hypothesis would help readers evaluate whether the study was sufficiently powered to detect theoretically meaningful effects.

We now supplement our approach of evaluating the (conservative) estimate of a typical effect size of interest with an evaluation of a smallest size of interest, which we define as half the magnitude of the typical effect size: (Methods). We used the relevant simple effect mean from Whitford et al. (2025) as an indicator of a typical effect size of interest and the simple effect mean divided by two as an indicator of the smallest effect size of interest.

The results of this additional analysis have been added to the Results section of the revised manuscript: (N1). From the posterior distribution (M = -0.3931, SD = 0.212), the probability that the current effect was larger than +0.64 µV (i.e., the mean of the Passive vs. Match contrast in the (Whitford et al., 2025) paper) was p < 0.001, while the probability that the current effect was larger than +0.32 µV (i.e., the smallest effect-size-of-interest, defined as half the simple effect mean described in Whitford et al., (2025)) was p<.001. N1-amplitude also did not differ significantly between the Primed condition (M = -5.158) and the Misprimed condition (M = 4.984) (t(69) = 0.892, p = .375). From the posterior distribution (M = -0.1741, SD = 0.200), the probability that the observed effect was larger than +0.39 µV (i.e., the mean of the Match vs. Mismatch contrast in the (Whitford et al., 2025) paper) was p = 0.002, while the probability that the current effect was larger than +0.2 µV (i.e., the smallest effect-size-of-interest, defined as half the simple effect mean described in Whitford et al., (2025)) was p = 0.032. (P2). From the posterior distribution (M = -0.1569, SD = 0.197), the probability that the observed effect was larger than +0.80 µV (i.e., the mean of the Passive vs. Match contrast in the (Whitford et al., 2025) paper) was p < 0.001, while the probability that the observed effect was larger than +0.40 µV (i.e., the smallest effect-size-of-interest, defined as half the simple effect mean described in Whitford et al., (2025)) was p = 0.0024.

13. Report effect sizes and uncertainty for the key pairwise contrasts in the main text. The manuscript reports means and p values for some contrasts, but it would help readers to see effect sizes and confidence intervals or credible intervals for Primed-Misprimed and Primed-Control differences for N1, P2, and pre-stimulus activity. Since the paper hinges on null or near-null effects, interval estimates are especially important.

We have added measures of effect size (Cohen's dz) and interval estimates (95% CIs) for the pairwise contrasts to the revised manuscript. The relevant additions are described below, and have been incorporated into the Results section of the revised manuscript:

N1 Primed vs. Control: Cohen's dz = 0.23, 95% CI for difference [-0.803, 0.017] N1 Primed vs. Misprimed: Cohen's dz = 0.11, 95% CI for difference [-0.563, 0.215] P2 Primed vs. Control: Cohen's dz = 0.10, 95% CI for difference [-0.538, 0.224] P2 Primed vs. Misprimed: Cohen's dz = 0.13, 95% CI for difference [-0.172, 0.553] Pre-Stimulus Primed vs. Control: Cohen's dz = 0.02, 95% CI for difference [-1.874, 1.640] Pre-Stimulus vs. Misprimed: Cohen's dz = 0.14, 95% CI for difference [-3.797, 1.024] 14. The near-significant Primed vs Control N1 difference deserves brief treatment. The N1 in the Primed condition was numerically more negative than in Control, with p = .06. I do not think this undermines the main conclusion, but it should be discussed because it runs opposite to the expected suppression direction and may indicate small unspecific effects of the task manipulation.

We have added the following few sentences to the relevant section of the Results:

Indeed, N1-amplitude was actually numerically greater (more negative) in the Primed condition (M = -5.158) relative to the Control condition (M = -4.765). Though this contrast did not reach statistical significance (t(69) = 1.92, p = 0.06), the mean difference may be suggestive of a small effect of the priming manipulation, but in the opposite direction to what we have observed in our previous inner-speech studies. In other words, this trend-level result provides, if anything, evidence against the claim that the inner speaking-induced suppression effect we have previously observed is due to a priming effect.

15. Similarly, clarify the rationale for the N1 quantification approach. N1 was quantified via peak-picking within 25-175 ms, whereas P2 used a windowed mean. Peak measures can be noisier and more sensitive to latency jitter. Please explain why peak-picking remains the preferred choice here, and consider adding a supplementary mean-amplitude analysis for N1 over a predefined window to show robustness.

We chose to use a peak-picking approach for the sake of consistency with our previous studies on which the current study is based. We typically use a peak-picking approach when analyzing the N1 component (e.g., Whitford et al., (2017) eLife, Whitford et al., (2025) eNeuro, Whitford et al., (2025), Schizophrenia Bulletin, etc.) though we have also used a time-window-based approach on occasion (e.g., Jack et al., (2019), NeuroImage). The N1 component is a large, early, clearly-defined component with a consistent latency and a consistent scalp topography. This component is thus well-suited for a peak-picking approach, and in our experience this approach tends to be slightly more sensitive at detecting significant effects compared to a time-window approach. However, we are happy to include an additional time-window-based analysis for the sake of robustness. This additional analysis is described below; as can be seen, the pattern of results was identical between the peak-picking and time-window-based approaches.

For the sake of robustness, we also analyzed the N1 data using a time-window-based approach. The N1 time-window was defined as 82-102 ms, based on the collapsed localizer approach (Luck and Gaspelin, 2017). The pattern of results was identical to the peak-picked data: the main effect of Condition was non-significant (F(2,138) = 0.572, p = .560, ηp2 = 0.008), and none of the pairwise contrasts approached significance (all ps > .1). For completeness, there was a main effect of Electrode (F(8,552) = 90.691, p<.001, ηp2= 0.568), but the Condition * Electrode interaction was non-significant (F(16, 1104) = 0.554, p=.638, ηp2 = 0.008).

16. Expand the interpretation of the 7-9 Hz cluster or temper it. At present, the theta/alpha cluster is used both as evidence for a priming-like effect and as a manipulation check. Both are plausible, but the current evidence is thin. The manuscript should either present this as exploratory or provide stronger grounding for why that frequency range and temporal extent should index priming/expectancy.

We now explicitly acknowledge the exploratory nature of this analysis in the following addition to the revised manuscript:

However, it should be noted that this analysis was exploratory. As such, the interpretations discussed above, while plausible, remain tentative and require replication in future research before firm conclusions can be drawn.

17. Please discuss block structure as a possible source of sustained-state effects. Blocks were organised by prime type ("Prime /ba/," "Prime /bi/," "Prime /fo/"). This may induce sustained context or habituation/adaptation effects beyond trial-wise priming. This concern could be addressed in future work by using randomised trial-by-trial cues and explicitly manipulating cue validity. I do not see this as fatal, but it should be acknowledged, particularly since the theoretical question concerns subjective expectation.

We now discuss block structure in the following addition to the revised manuscript:

A notable feature of the experimental design is that the blocks were organized by trial-type; that is, participants completed 24 'Prime /ba/' trials (for example), followed by 24 'Prime /bi/' trials, etc. We used this 'blocked' approach for consistency with the previous studies on which the current study is based. However, a potential limitation of this approach is that it opens up the possibility of habituation / adaptation effects developing across the course of the block. An alternative approach would have been to change the trial-type every trial. While such a 'trial-by-trial' approach would have avoided the possibility of habituation / adaptation across the block, it could potentially have led to other challenges, such as difficulty maintaining concentration to ever-changing task instructions (and would have created a confound when attempting to compare results with previous findings). Nevertheless, replicating these results using a trial-by-trial design would be a worthwhile aim for future research.

18. Regarding Figure 2, Panel E appears to suggest potential differences between conditions that are not reflected in the reported statistical results. The authors may wish to verify that the plotted data and exported values are consistent, and clarify whether any visualization choices (e.g., scaling or averaging procedures) could account for this apparent discrepancy.

We can confirm that Figure 2, Panel E is correct. The reason why the 'Misprimed' waveform for the CNV appears higher (more positive) than the Primed and Control waveforms is driven by two participants (ID numbers 23710 and 23713) who are clearly visible as outliers in Figure 2, Panel F), and who showed an unusual pattern of pre-stimulus activity. Specifically, rather than the expected negative-going (or flat) pre-stimulus waveform, these two participants showed a large, positive-going waveform that continued to elevate throughout the entire pre-stimulus period. It should be emphasized that even with these two outliers included (as is currently the case), the between-condition ANOVA did not approach significance, and the Misprimed condition did not differ statistically from zero. This pattern of results did not change if these two outliers were removed from the analysis.

We have added a discussion of this issue to the revised manuscript, as follows: (As an aside, it is notable that in Figure 2E the Pre-stimulus waveform appeared somewhat higher (i.e., more positive) for the Misprimed condition compared to both the Primed and Control waveforms. This apparent difference was driven by two participants (visible as outliers in Figure 2F) who showed an unusual pattern of pre-stimulus activity; specifically, these participants showed a large, positive-going waveform that continued to elevate throughout the pre-stimulus period. It should be emphasized that this between-condition difference in CNV did not approach statistical significance with these participants included (p = .359). Moreover, we verified that excluding data from these two outlying participants did not change the pattern of significant and non-significant findings in any of the reported analyses).
